# Supplementary material for: Operando spectroscopy study of the carbon dioxide electro-reduction by iron species on nitrogen-doped carbon
Source: Nat Commun. 2018 Mar 5;9:935. doi: 10.1038/s41467-018-03138-7 (PMC5838105; doi:10.1038/s41467-018-03138-7)
Supplement: Supplementary file 1 — Supplementary Information [file 41467_2018_3138_MOESM1_ESM.pdf]

SUPPLEMENTARY FIGURES

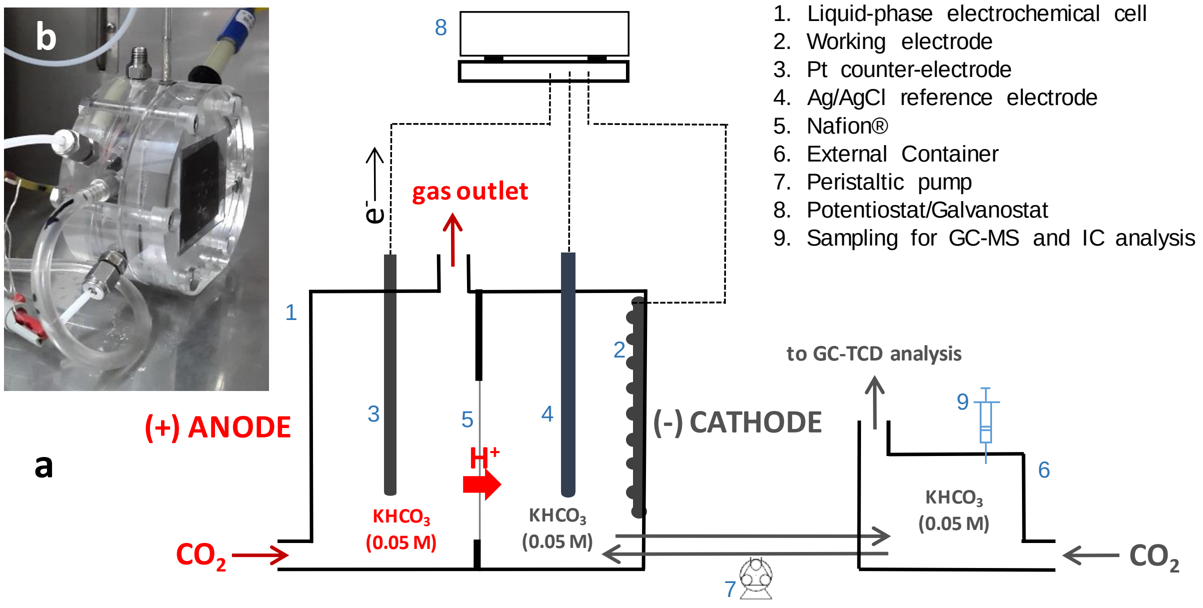

Supplementary Figure 1. Electro-catalytic device for CO_2 reduction **a** Schematic drawing of the experimental cell with details of electrodes, charge pathways and electrolyte **b** picture of the cell. Adapted from reference [1]

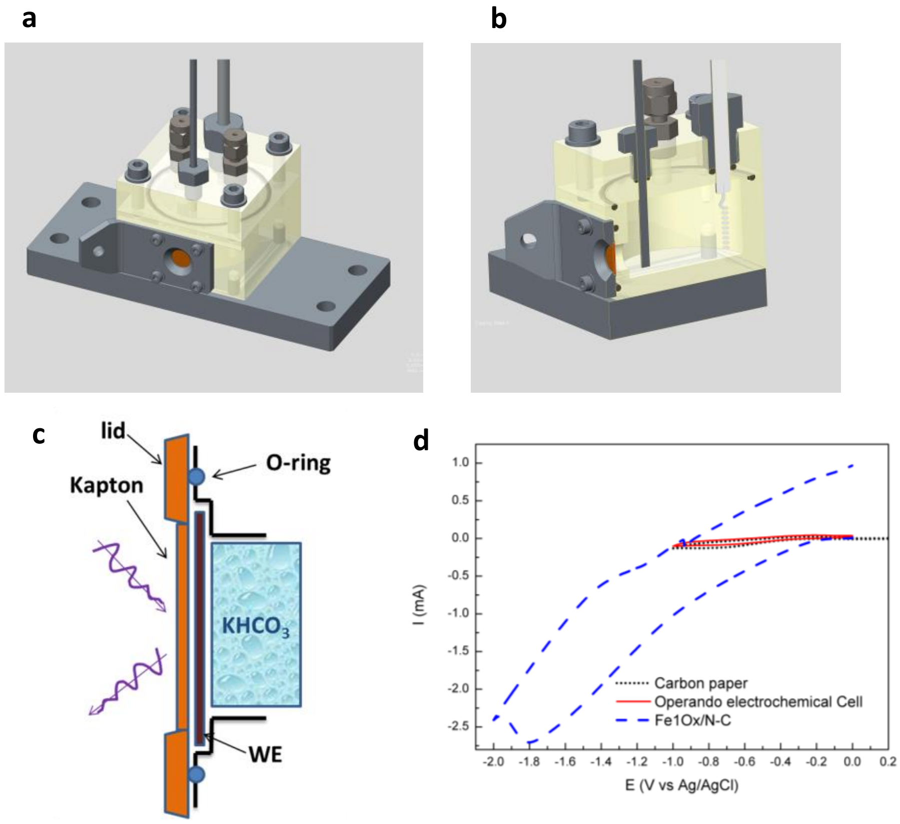

Supplementary Figure 2. Electrochemical cell for Operando XAFS study **a** Front view of the electrochemical cell for Operando XAFS study designed for B18 beamline at DLS; **b** cross section; **c** detail of the WE sample position. **d** A comparison of the CV (cyclic voltammetry) of cell, carbon paper and Fe/N-C. The images in **a** and **b** belongs to Diamond Light Source Limited and is not included in the CCBY license. Diamond is a not-for-profit limited company funded as a joint venture by the UK Government through the Science & Technology Facilities Council (STFC) in partnership with the Wellcome Trust.

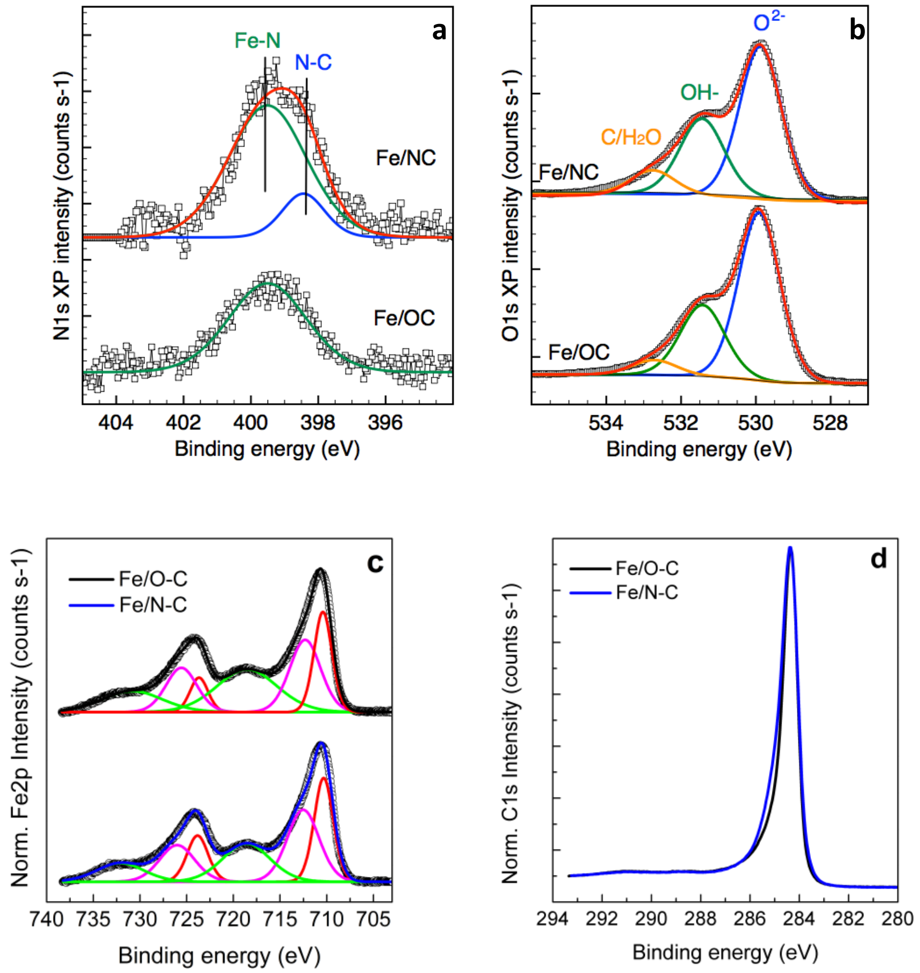

Supplementary Figure 3. XPS spectra of Fe/N-C and Fe/O-C samples **a** Deconvolved N1s XP spectra for Fe/N-C and Fe/O-C in UHV at KE 450 eV. Fitting: N-C (398.4 eV) is pyridine-like N substituted at the edge of the graphite planes (component in blue) [2]. Fe-N (399.6 eV) is the component which includes all the types of Fe-N bonds originating from the $Fe(NO_3)_3$ precursor (component in green). **b** Deconvolved O1s XP spectra for Fe/N-C and Fe/O-C at KE 450 eV. Fitting: O^{2-} species (530 eV) is bridge-oxygen bound to Fe^{3+} cations of the ferrihydrite structure; OH species (531.5 eV) are terminal species bound to Fe^{3+} cations of the ferrihydrite structure; chemisorbed water (533 eV). **c** Deconvolved Fe2p spectra for Fe/N-C (blue line) and Fe/O-C (black line) considering two components at 710 eV and 712.14 eV and the satellite feature at 718.8 eV, characteristic of $Fe^{(III)}$ oxide. The ΔBE of the spin orbit splitting is 13.5 eV. The spectra resemble the spectrum reported for FeOOH in reference [2]. **d** C1s spectra for Fe/N-C (blue line) and Fe/O-C (black line). Note the more symmetric C1s peak of the Fe/N-C and the higher intensity above the main graphitic component at 284.4 eV characteristic of N functionalities [3].

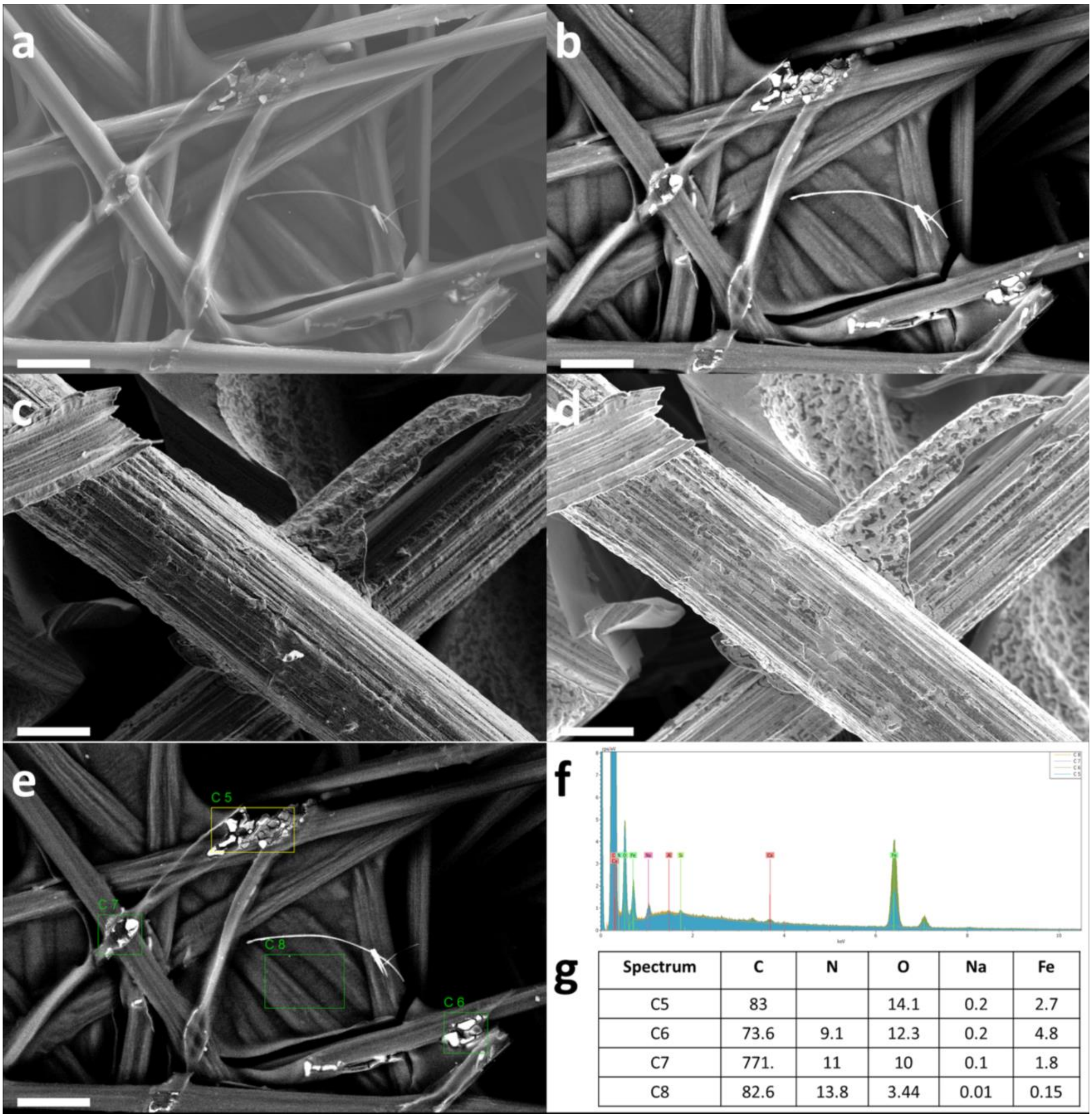

Spectrum	C	N	O	Na	Fe
C5	83		14.1	0.2	2.7
C6	73.6	9.1	12.3	0.2	4.8
C7	771.	11	10	0.1	1.8
C8	82.6	13.8	3.44	0.01	0.15

Supplementary Figure 4. SEM characterization of Fe/N-C sample **a** Secondary electron (SE) image. Scale bar 20 μm. **b** Backscattered electron (BSE) image. Scale bar 20 μm. **c** High magnification SE and **d** BSE SEM images. Scale bar 4 μm. **e** Low magnification BSE SEM image. Scale bar 20 μm. **f** EDX spectra and **g** corresponding quantitative analysis at several spots (C5-C8) in **e** as indicated therein.

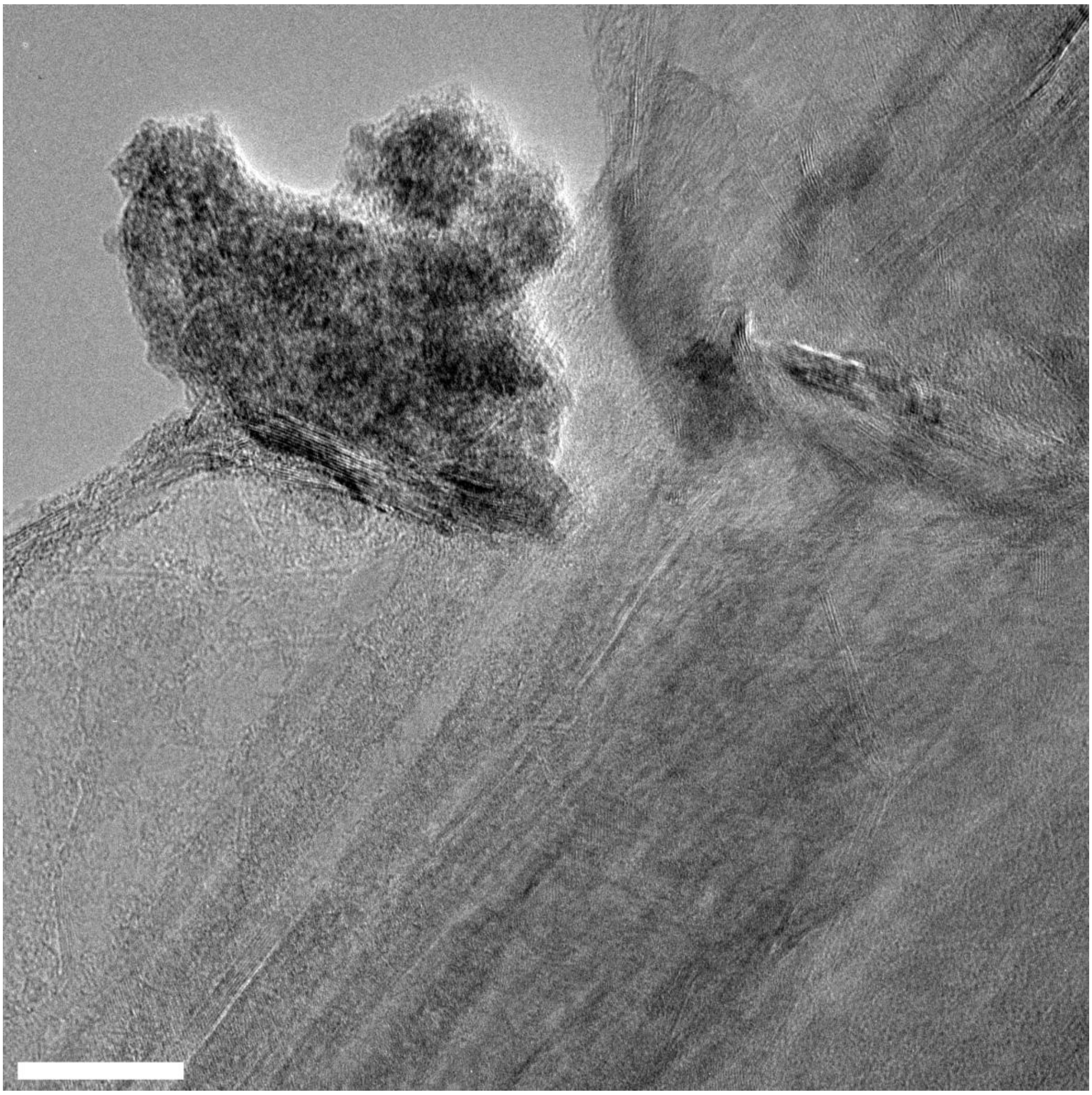

Supplementary Figure 5. HRTEM image of sample Fe20/O-C (20% weight Fe loading). Scale bar 20 nm.

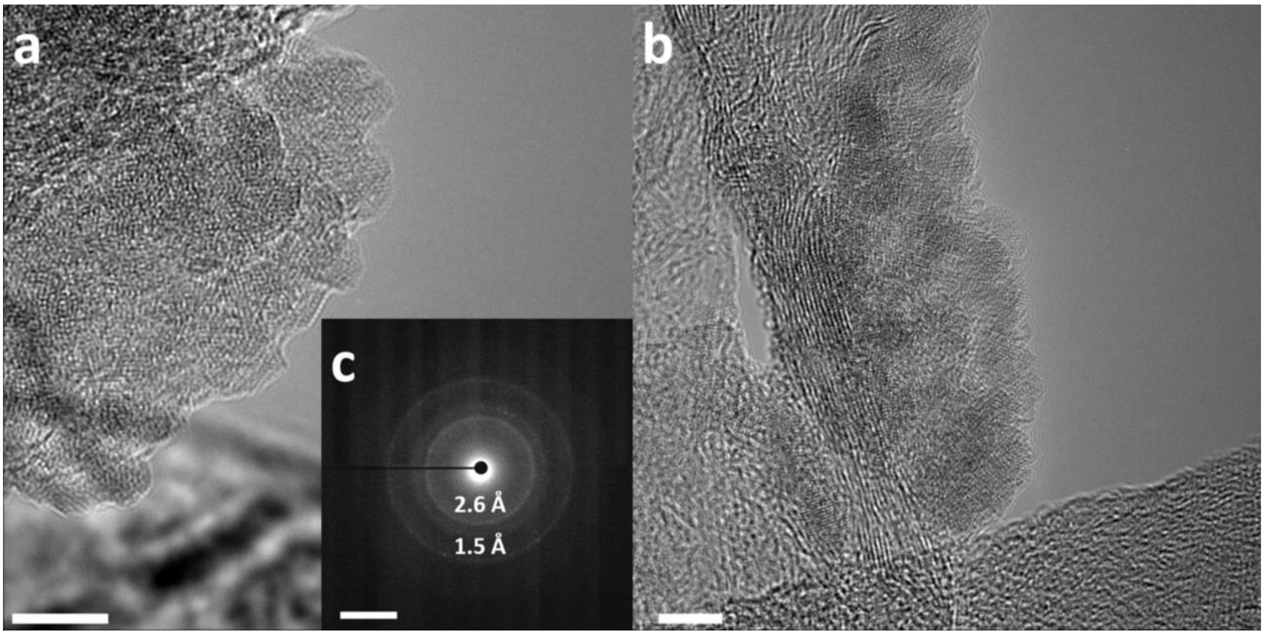

Supplementary Figure 6. Nanostructure of sample Fe/N-C **a** and **b** HRTEM images. Scale bar 5 nm. **c** Selected area electron diffraction pattern exhibiting two rings, corresponding to distances of 0.15 and 0.26 nm. [4] Scale bar 1 1/nm.

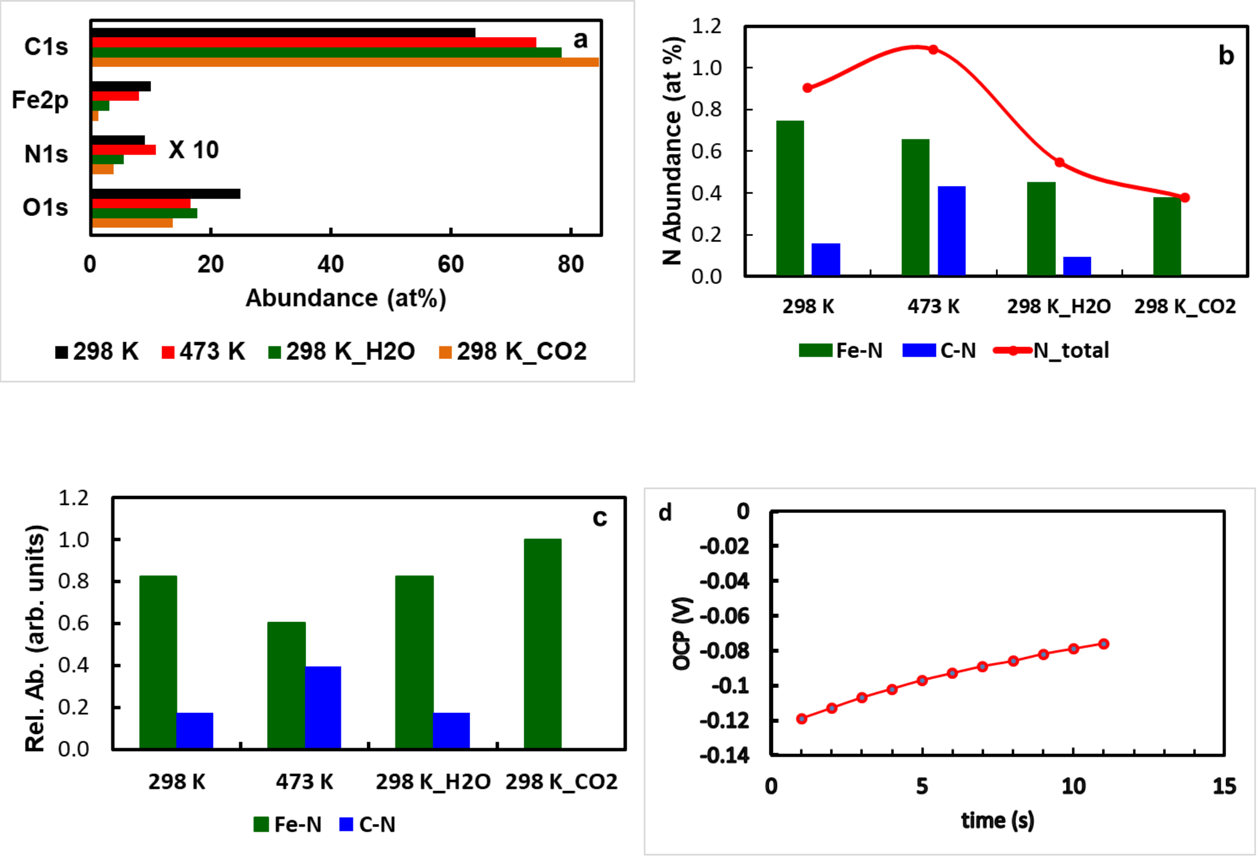

Supplementary Figure 7. Structural and elemental composition dynamics for Fe/N-C sample studied by ambient pressure XPS **a** Absolute atomic abundance of the elements at different experimental conditions applied in the following order: UHV and 298 K (black); UHV and 473 K (red); 0.15 mbar H_2O and 298 K (green); 0.15 mbar CO_2 and 298 K (orange) after evacuation of the chamber to 10^{-7} mbar. Note that after dosing the water, the chamber was evacuated to a pressure of 10^{-7} mbar in order to remove most of the chemisorbed water **b** Absolute atomic abundance of the different N components of the fitted N1s XP spectra at the different experimental conditions. **c** Relative abundance of the different N components of the fitted N1s XP spectra at the different experimental conditions. XP spectra were recorded collecting photoelectrons emitted with KE=150 eV corresponding to a depth of information of about 0.5 nm. Any changes of the N species due to their thermal decomposition can be disregarded because only a negligible amount of N is desorbed in the gas phase as NO molecules and measured by mass spectrometry. **d** Open circuit potential (OCP) as function of time measured for the sample Fe/N-C upon immersion in 0.05M $KHCO_3$.

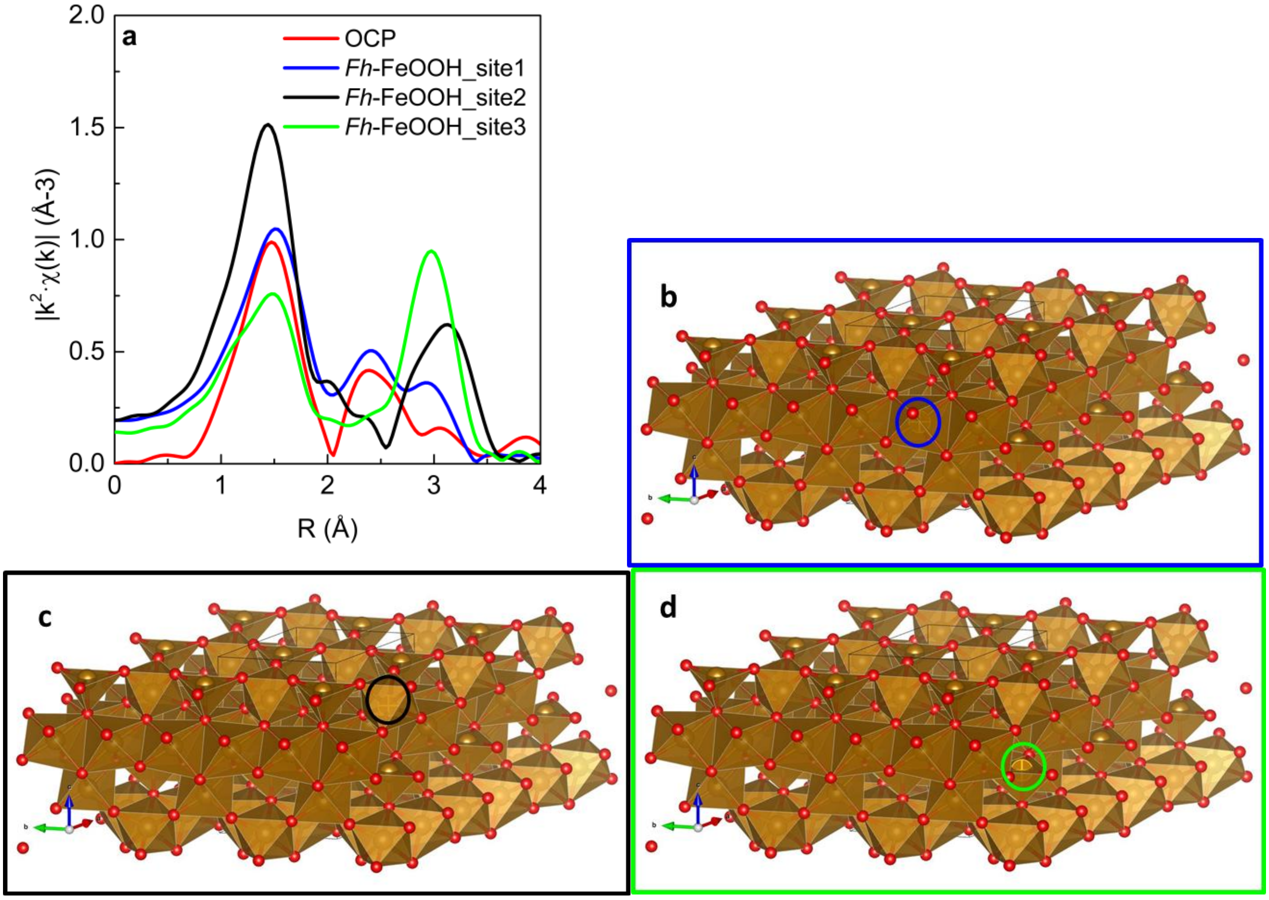

Supplementary Figure 8. Simulated and experimental FT-EXAFS spectra of ferrihydrite **a** FT-EXAFS spectrum measured at OCP in 0.05M $KHCO_3$ (red line) and simulated FT-EXAFS spectra for Fe site 1 (blue line) Fe site 2 (black line) and Fe site 3 (green line) in the *Fh*-FeOOH structure using Artemis software (IFEFFIT package) Feff6.0 code. Model of ferrihydrite structure imported from indicated cif file using VESTA software [5] with indication of site 1 in **b**, site 2 in **c** and site 3 in **d**.

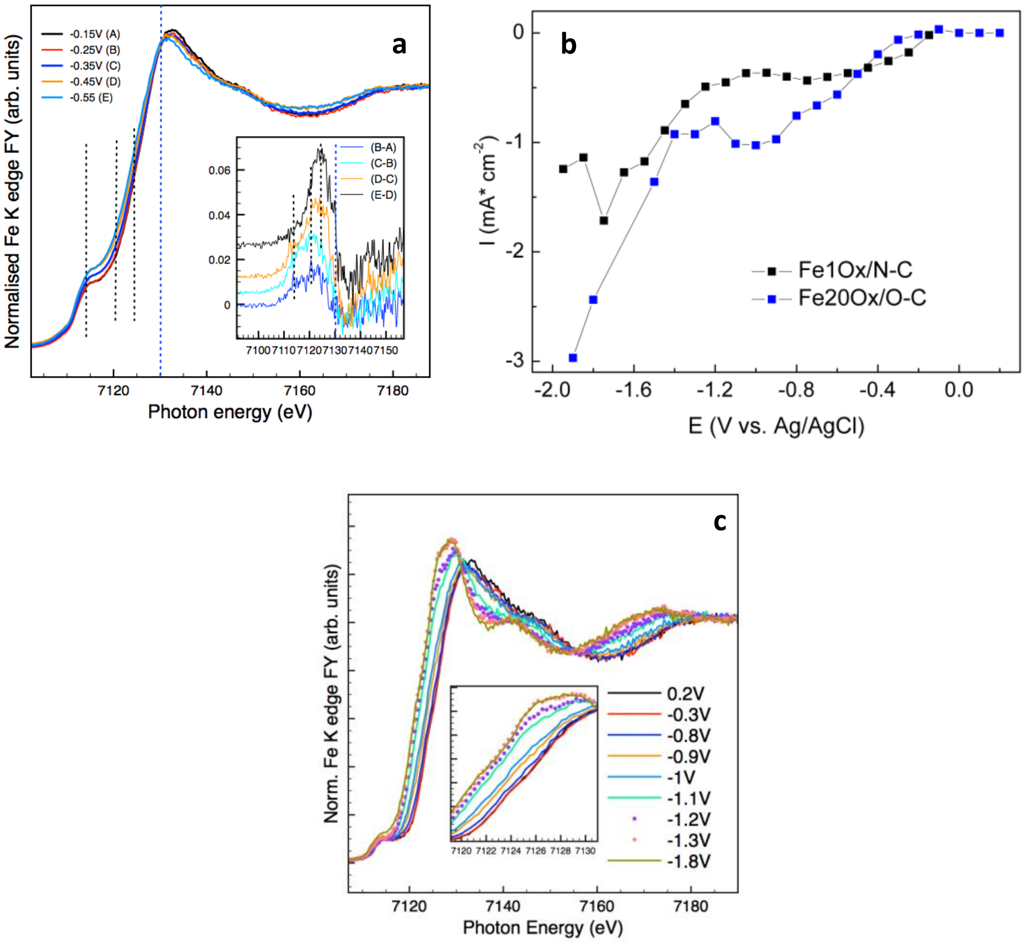

Supplementary Figure 9. FY-mode fast-XANES spectra measured for the sample Fe/N-C and Fe20/O-C *in operando* **a** fast-XANES Fe K edge XANES spectra measured in the CO_2RR selective potential region during the CA experiments and different spectra in the inset. **b** Instantaneous current measured at constant potential (CA) is reported as function of the potential itself for the fast-XANES measurements reported for Fe/N-C sample in Fig. 8 and for Fe20/O-C sample in c. **c** Fast-XANES Fe K edge spectra measured for the sample Fe20/O-C (20% weight Fe loading) in 0.05M $KHCO_3$ at different potential as indicated.

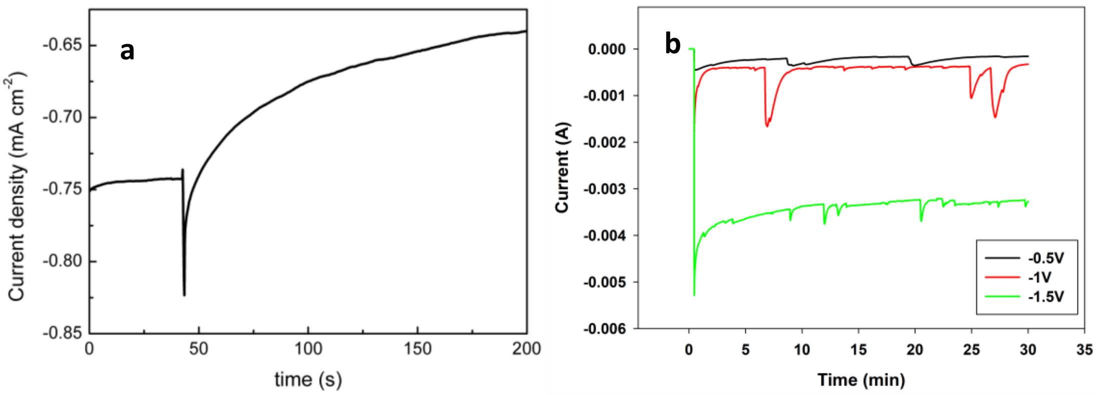

Supplementary Figure 10. Current profile at constant potential for Fe/N-C **a** Current profile during operando XAFS at a potential of -1.65 V. **b** Current profiles as a function of time during CA in liquid-phase at -0.5 V, -1 V and -1.5 V.

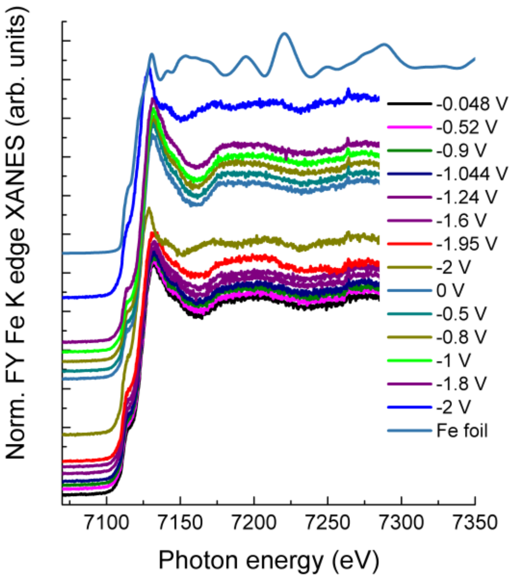

Supplementary Figure 11. Fast-XANES spectra measured for the sample Fe/O-C *in operando*
FY-mode Fe K edge XANES spectra measured in 0.05M $KHCO_3$ at different potential (as indicated) for the Fe/O-C. Each spectrum is merged from 11 fast Fe K-edge XANES spectra. Each spectrum is edge jump normalised and then an offset in the y axis is applied for a better representation. During the experiment, the potential was varied in steps of 0.1 V and hold at constant potential for 3 minutes. The plots show the reproducible redox processes that the Fe/O-C undergoes upon polarization.

SUPPLEMENTARY TABLES

Supplementary Table 1. Fitting parameters for Fe K-edge EXAFS spectra of Fe/O-C and Fe/N-C samples [a] using scattering paths calculated from a cif file PDF 00-058-0898.of ferrihydrite (PDF-4+ structure)

Sample	Abs. Sc.	N	R /Å	2σ2 / Å2	E0 / eV	R factor
Fe/N-C	Fe-O	3.6(6)	1.95(3)	0.003(set)	0.4±1.6	0.017
	Fe-O	1.9(6)	2.12(3)	0.003(set)		
	Fe-Fe	2.7(8)	3.05(3)	0.009(set)		
Fe/O-C	Fe-O	3.6(7)	1.95(30	0.003(set)	0.7±1.5	0.014
	Fe-O	1.9(7)	2.11(7)	0.003(set)		
	Fe-Fe	2.9(8)	3.05(3)	0.009(set)		

[a] Using $S0^2$ = 0.73, 1.8<k<10.6, 1<R<3, No. independent points =10, from reference [6]

Supplementary Table 2. Surface elemental composition determined by XPS in at% [a].

Sample	O	N	Fe	C	Fe-N	C-N
Fe/N-C	8.5	0.5	2.3	88.7	0.4	0.1
Fe/O-C	11.5	0.4	3.3	84.8	0.4	0

[a] The spectra were recorded by collecting photo-electrons with KE=450 eV, corresponding to a depth information of 1.5-2 nm. [7]

Supplementary Table 3: CO_2RR behaviour of Fe/N-C (Fe 20 wt. % loading) sample at -0.5 V, -1 V, -1.5 V vs Ag/AgCl

		Faraday Efficiency (%)			
Voltage, V	HCOOH	CH_3COOH	H_2	CO_2RR (a)	Total (b)
-0.5	2.1	0	18.1	2.1	20.2
-1	2.7	0	9.4	2.7	12.1
-1.5	0.2	0	99.7	0.2	99.9

(a) CO_2RR Faradaic efficiency. (b) CO_2RR and HER Faradaic efficiency.

Supplementary Table 4: CO_2RR behaviour of Fe/O-C (Fe 20% weight loading) sample at -0.5 V, -1 V, -1.5 V vs Ag/AgCl

	Faraday Efficiency (%)				
Voltage, V	HCOOH	CH_3COOH	H_2	CO_2RR (a)	Total (b)
-0.5	5.7	0	48.1	5.7	53.8
-1	1.4	0	40.7	1.4	42.1
-1.5	0.1	0	50.9	0.1	51

(a) CO_2RR Faradaic efficiency. (b) CO_2RR and HER Faradaic efficiency.

SUPPLEMENTARY NOTES

Supplementary note 1:

Description of the Operando electrochemical Cell and details about XAFS experiments

The home-built electrochemical cell was specifically designed for the Core XAFS beamline B18 of the synchrotron facility Diamond Light Source and is equipped with a commercial Ag/AgCl microreference electrode (MI-409F) and a custom-made Pt spiral wire as counter electrode CE (Supplementary Figure 2a and b), both supplied by *microelectrods.inc*. The body of the cell is made of PTFE and presents an aperture on the front side hosting the working electrode WE (~1 cm^2), which is directly in contact with the liquid electrolyte. The cell is filled with 10 ml of a 0.05 M $KHCO_3$ solution, equal to a 2/3 of the total cell volume. The WE (indicated in Supplementary Figure 2c as a brown area) is placed in a recess area on the front wall of the cell and it is in electric contact with the lid (glassy carbon or stainless steel), which functions as current collector. The lid has a kapton window to avoid spilling of liquid on the one hand; on the other hand, it allows X-rays in and out. The leak-tightness is realized through an o-ring as indicated in the drawing (Supplementary Figure 2c). The thickness of the recess area is specifically designed to allow the sample to be in electric contact with the lid/current collector and at the same time to prevent the liquid to by-pass the sample. Indeed, the liquid accesses the front side of the sample through the porosity of the carbon paper such that only a very tiny volume of liquid is allowed between the sample and the kapton foil. A potentiostat was used to control the potential of the WE, while measuring the current between WE and CE. The electrochemical cell was tested against a standard electrochemical cell in glass equipped with a Pt wire CE and a Ag/AgCl reference electrode. The electrochemical behaviour of Au foil in 0.5M H_2SO_4 was used as comparison of the performance of both cells, which provided the same cyclic voltammetry (CV) results (not shown). Preliminary electrochemical tests on the electrocatalysts showed consistent results with the bench electrochemical tests.

A comparison of the CV of cell, carbon paper and Fe/N-C is reported in Supplementary Figure 2d.

For the operando experiments, first, XAFS measurements were performed for 30 minutes (10 repetitions, 3 minutes each spectrum) on the dry sample mounted on the cell and afterwards in 0.05M $KHCO_3$ at OCP also for 30 minutes. The spectra of the dry sample and in $KHCO_3$ were identical during the overall 60 minutes of measurement, ruling out any X-ray beam induced damage of the sample. Following that, cyclic voltammetry (CV) in 0.05M $KHCO_3$ was conducted on the electrocatalysts at a scan rate of 10 mVs^{-1}, in the voltage range from open circuit potential to -2 V and then to 0 V, and for several cycles until constant

behavior, attained already from the second cycle onward. Simultaneously XAFS measurements were performed as described previously during the 1st cycle.
Subsequently, chronoamperometry was also performed with simultaneous quick-XANES measurements as described previously. The potential was hold for 3 minutes and then was stepwise increased from OCP to -2 V, in steps of 0.1 V each.

Supplementary note 2

Operando XAFS and corresponding electrochemical behaviour of Fe/N-C sample

Supplementary Figure 9a shows the Fe K edge spectra measured during the CA experiments between -0.15 V and -0.55 V vs Ag/AgCl. This is the CO_2RR selective potential region. The difference spectra in the inset indicate a downshift of the edge. In Supplementary Figure 9b, the instantaneous current measured at constant potential (CA) is reported as function of the potential itself for the quick-XAFS measurements of Figure 8 of the main text.

During the operando study, at more negative potential, when HER is the main reaction path, the catalyst's instability manifested itself as a sharp cathodic current peak (Supplementary Figure 10a). Indeed, this is a characteristic behaviour of these catalysts, which was also observed during the bench electrochemical tests, more markedly at more negative potential (Supplementary Figure 10b). Such sharp current peaks were previously accounted for as a result of the leaching of metal species into the solution, or to the formation of gas bubbles sticking on the catalysts surface and their subsequent release. [8] We believe that certainly the latter phenomenon takes place in these experiments because we also observe a reduction of the fluorescence signal intensity at the point of maximum current. This suggests that the growth of H_2 bubbles increases the thickness of the medium that the incoming and emitted X-rays travel through resulting in a reduced transmission. The intensity is however immediately restored as the bubbles are released and diffuse away from the electrode surface. The corresponding catalyst structural dynamics indicate that from an oxidized Fe-L species the catalyst becomes metallic at the point of maximum current. The catalyst undergoes several turnovers between these two states starting from -1.25 V with non-defined time dependence. The oxidized Fe-L species which converts into Fe^0 upon H_2 release is characterized by a very intense resonance at ca. 7130 eV.
Despite the presence of O species observed in the Fourier transform EXAFS spectrum relative to this species (Figure 8b in the main text), such high intensity implies a different chemical environment than the Fe(II)-O species observed at less negative potential. A similar pronounced increase of the white line intensity was observed for Fe-Fe hydrogenase upon hydride bond formation and protonation.[9] Analogously, we can assume a similar situation occurs here.

In alkaline solution, the HER occurs from H^+ discharge.[10] Therefore one would assume that in the electrical double layer, H^+ species accumulate and chemisorb at more negative potential. As a consequence of the

higher e^- availability, the Fe(II)-L could be related to the formation of hydride species, which may originate from the dissociation of chemisorbed OH. We hypothesize that the dynamics observed are related to the discharge of H^+ leading to HER, via interaction with intermediate hydride species. Despite the mechanism is unclear, the results indicate that HER occurs on a metallic surface, which implies that, the CV previous to the bulk electrolysis is not an “innocent” treatment and can modify the selectivity of the electro-catalyst.

SUPPLEMENTARY REFERENCES

1. Ampelli, C. et al. Electrocatalytic Conversion of CO_2 to Produce Solar Fuels in Electrolyte or Electrolyte-Less Configurations of PEC Cells. *Faraday Discuss.* **183**, 125–145 (2015).

2. Grosvenor, A.P. et al. Investigation of muktiplet splitting of Fe2p XPS spectra and bonding in iron compounds. *Surf. interface Anal.* **36**, 1564–1574 (2004).

3. Arrigo, R. et al. Tuning the Acid/Base Properties of Nanocarbon by Functionalization via Amination. *J. Am. Chem. Soc*. **132,** 9616–9630 (2010).

4. Weatherill, J. S. et al. Ferrihydrite formation: the role of Fe13 Keggin clusters. *Environ. Sci. Technol.* **50**, 9333–9342 (2016).

5. Momma, K. & Izumi, F. VESTA 3 for three-dimensional visualization of crystal, volumetric and morphology data. *J. Appl. Crystallogr*. **44**, 1272–1276 (2011).

6. Forde, M. M. et al. Light alkane oxidation using catalysts prepared by chemical vapour impregnation: Tuning alcohol selectivity through catalyst pre-treatment. *Chem. Sci*. **5**, 3603–3616 (2014).

7. Arrigo, R. et al. Dynamic of Pd on NCNT in the Direct Synthesis of H_2O_2. *ChemSusChem* **7,** 179–194 (2014).

8. Wang, M., Wang, Z. & Guo, Z. Understanding of the intensified effect of super gravity on hydrogen evolution reaction. *Int. J. Hydrog. Energy* **34,** 5311–5317 (2009).

9. Löscher, S. Schwartz, L. Stein, M. Ott, S. & Haumann, M. Facilitated Hydride Binding in an Fe-Fe Hydrogenase Active-Site Biomimic Revealed by X-ray Absorption Spectroscopy and DFT Calculations. *Inorg. Chem.* **46**, 11094–11105 (2007).

10. Awad, S. A. Discharge of Hydrogen Ions at Metal Cathodes in Alkaline Solutions. *Chem. Papers*. **48**, 5–6 (1994).
